# Fluorescent Sensors Based on Organic Polymer-Capped Gold Nanoparticles for the Detection of Cr(VI) in Water

**DOI:** 10.1155/2019/1756014

**Published:** 2019-02-03

**Authors:** Na Wang, Liangchen Wang, Hong Yang, Tingting Xiong, Shangping Xiao, Jiawen Zhao, Weiping Du

**Affiliations:** School of Chemistry & Chemical Engineering, Southwest Petroleum University, Chengdu 610500, Sichuan, China

## Abstract

“Turn-off” fluorescent sensors for Cr(VI) have been fabricated based on organic polymer-capped gold nanoparticles. The fluorescence intensity, as well as the response behavior of the sensors, is dependent on the pH values of buffer solution and dilution amounts of the sensors. When diluted 50 times with pH 2.0 buffer solution, the sensors show good linear responses toward Cr(VI) at concentrations between 2.8–5.9 *μ*M and 5.9–29 *μ*M. The calculated detection limit is 0.63 *μ*M (S/N=3). The interference study and real sample assays exhibit satisfying selectivity and reliability results. Furthermore, the quenched intensity of fluorescence could be recovered by Fe(II) ion, which provides a potential method to detect Fe(II) ions. The quenching and recovering mechanisms have also been investigated. It is suggested that the quenching mechanism is based on the combined effects of internal electron transfer and the inner filter effect. Finally, the recovering mechanism is based on the redox reactions between the Cr(VI) and Fe(II) ions.

## 1. Introduction

Chromium is commonly used in industrial applications, such as producing many commercially important alloys and tanning agents. Although chromium is regarded as an essential trace element for animals and humans [1], its harmful effects to the environment are arguably more familiar to people than its potential applications. The toxicity of chromium compounds differs from each other. Cr(0), Cr(III), and Cr(VI) are three thermodynamically stable Cr forms in the environment, but only Cr(VI) exhibits remarkably high toxicity toward animals and humans when compared to Cr(0) and Cr(III) [2]. According to reports by the American Conference of Governmental Industrial Hygienists (ACGIH), the exposure limit of Cr(VI) is one-tenth of Cr(III) compounds [3]. The occupational exposure to Cr(VI) compounds leads to harmful skin, nasal, and renal effects as well as hepatic lesions. Furthermore, Cr(VI) compounds are carcinogenic, bringing about respiratory cancer in humans and animals [4–6]. Considering Cr(VI) is a persistent contaminant in many water sources, it is vital to monitor Cr(VI) content in the environment.

There are already a wealth of detection methods used to detect chromium ions in water, including atomic absorption spectroscopy (AAS) [7, 8], inductively coupled plasma atomic emission spectroscopy (ICP-AES) [9, 10], electrochemical methods [11, 12], colorimetry [13, 14], and fluorescence spectroscopy [15–17]. However, among these methods, AAS and ICP require expensive and cumbersome instruments and are difficult to use for speciation determination of ions. Meanwhile, electrochemical and spectrochemical methods often demand complex preparation procedures and are susceptible to interference from sample matrices. These issues necessitate the exploration of novel and efficient methods for the detection of Cr(VI). During the past decade, fluorescent sensors have become an important research field of biochemical and environmental analyses and have attracted great attention because of their facility, sensitivity, and selectivity in fluorescent assays. Small organic molecules, inorganic quantum dots, heavy metal nanomaterials, and polymers have made a profound impact and have endowed modern sensory systems with superior performance. Among those sensors, the polymer-based sensors have received great attention due to their amplification of signals, facile fabrication into chips or microarrays, multifunctional outputs, and other benefits [18, 19].

Polyethyleneimine (PEI) is a cationic, water-soluble polymer with abundant amino groups. Several reports have unraveled that the amino groups of PEI and aldehydes (or ketones) can react to produce fluorescent polymers [20, 21], of which the fluorescence originates from the Schiff base bonds [20]. This fluorescent Schiff base polymer and the noble metal nanoclusters modified by it have been prepared to fabricate the fluorescent sensors. These sensors show high sensitivity and selectivity toward the target subjects [16, 22–24]. In this work, we have prepared formaldehyde-modified, hyperbranched polyethyleneimine- (F-hPEI-) capped Au NPs by one-pot synthesis. The emission fluorescence of the sensors was selectively quenched by Cr(VI) because of the strong and specific reaction between the Cr(VI) and the sensor. To the best of our knowledge, Cr(VI) sensors based on F-hPEI-capped Au NPs have not been reported.  Table S1 (Supplementary Materials) shows the comparison of the proposed sensor with other reported methods for the detection of Cr(VI) in aqueous samples. The limit of detection (LOD) value of our sensors is not extremely sensitive, but is still competitive. It is lower than 0.05 mg/L (0.96 *μ*M), which is the total chromium guideline value for drinking-water quality set up by the World Health Organization (WHO) [25]. Furthermore, the sensor also provides a method for the speciation of Cr(VI) and Cr(III), which is a benefit that is not present in other AAS or ICP approaches. As a result, the proposed sensor is an alternative efficient method for Cr(VI) determination and speciation. The fluorescence and UV-Vis spectra illustrate the sensor is “turned off” by Cr(VI) due to the combined effects of internal charge transfer (ICT) and the inner filter effect (IFE). The proposed sensors show satisfying sensitivity and reliability toward the determination of Cr(VI). Furthermore, the recovery of the fluorescence intensity can be realized through the redox reaction between Cr(VI) and Fe(II) ion. It is a new “turn-off” fluorescent sensor for Cr(VI) determination, which not only provides a quantitative approach but also offers the speciation of Cr(VI) and Cr(III) as well as Fe(III) and Fe(II).

## 2. Materials and Methods

### 2.1. Materials

All of the chemicals used were of analytical grade. Hyperbranched polyethyleneimine (hPEI, Mw= 10000, 99%) was obtained from Aladdin Industrial Corporation (Shanghai, China), and HAuCl_4_·3H_2_O (≥49.0% Au basis) was bought from Sigma-Aldrich (Shanghai, China). Quinine sulfate dehydrate (99.0%) was obtained from Aladdin Industrial Corporation (Shanghai, China). Cr certified reference material (CRM) was bought from Guobiao Testing & Certification Co., Ltd. (Beijing, China), of which the concentration was 1000 *μ*g/mL. Formaldehyde (35 wt%), K_2_Cr_2_O_7_ (≥99.8%), (NH_4_)_2_Fe(SO_4_)_2_·6H_2_O (≥99.7%), CrCl_3_ (99.0%), Fe_2_(NO_3_)_3_·H_2_O (≥98.0%) CuSO_4_·5H_2_O (≥99.0%), KH_2_PO_4_ (≥99.5%), K_2_HPO_4_·6H_2_O (≥99.5%), H_3_PO_4_ (≥85 wt% in H_2_O), NaOH (≥98.0%), HCl (36%-38%), HNO_3_ (65%-68%), NaCl (≥99.5%), NaBr (≥99.0%), NaHCO_3_ (≥99.5%), NaClO_3_ (99.0%), KI (99.0%), KCl (≥99.5%), Mg(NO_3_)_2_ (≥99.0%), CaCl_2_ (≥96.0%), MnCl_2·_4H_2_O (≥99.0%), Zn(NO_3_)_2_·6H_2_O (≥99.0%), Ni(NO_3_)_2·_6H_2_O (≥98.5%), Cd(NO_3_)_2_·4H_2_O (≥99.0%), Al_2_(SO_4_)_3_·18H_2_O (≥99.0%), AgNO_3_ (≥99.8%), Bi(NO_3_)_3_ (≥99.0%), and Pb(NO_3_)_2_ (≥99.0%) were supplied by Chengdu Kelong Chemical Reagent Plant (Sichuan, China). The buffers used were potassium phosphate monobasic/HCl (pH ≤ 4), potassium phosphate monobasic/NaOH (8 ≥ pH >4), and potassium phosphate dibasic/NaOH (pH ≥ 9). Phosphate-buffered solutions from 1.0 to 11.0 were prepared according to standard protocols and the pH values were confirmed with a PHS-25 pH meter (Leici, China). The buffer solutions used in this study were 50 mM at various pH values. All glassware was cleaned with fresh aqua regia (HCl/HNO_3_ = 3:1, v/v) before use. The water used in all the experiments was ultrapure water.

### 2.2. Instrumentation

Fluorescence measurements were performed on a fluorescence spectrophotometer (Hitachi, F-7000, Japan). Fluorescence lifetime was measured on a Fluorolog-3 spectrofluorometer (Horiba JobinYvon) with a DeltaDiode (280 nm, DD-320, Horiba Scientific) as the excitation source and a picosecond photon detection module (PPD-850, Horiba Scientific) as the detector. The UV-Vis absorption spectra of the samples were obtained with a UV-Vis spectrophotometer (Ocean Optics, QE65 Pro, USA). The morphology of the nanoparticles was determined using a transmission electron microscope (TEM) (Libra200, Carl Zeiss, Germany). The samples were deposited onto carbon-coated copper grids and were dried in air at room temperature for TEM analysis. The real water samples were analyzed using a NexION 350X ICP-MS spectrometer (PerkinElmer, USA), while the spiked water samples were determined by an atomic absorption spectrophotometer (AA-7020, East & West Analytical Instruments, China) for comparison with the proposed sensor.

### 2.3. Synthesis of F-hPEI-Capped Au NPs

F-hPEI-capped Au NPs were synthesized according to the literature [24]. Briefly, 4 mL 0.05 g/mL hPEI, 5.8 mL of H_2_O, and 10 mL of HAuCl_4_ (0.01 M) were added to a flask sequentially and stirred for 2 min. Then, 0.2 mL of formaldehyde (35 wt%) was added to a flask. The mixture was refluxed for 1 h at 90°C. The products were simply purified by dialysis through a porous cellulose bag (molecular weight cut off 5000 Da) using ultrapure water for 24 h. The aqueous inside the dialysis bag was collected and stored in a refrigerator at 4°C.

### 2.4. Measurement Procedure

Briefly, 960 *μ*L of buffer solution, 20 *μ*L of F-hPEI-capped Au NPs, and 20 *μ*L of different concentrations of Cr(VI) were mixed and stirred vigorously. The mixed solution was then poured into a quartz cuvette. After 10 min, the fluorescence spectra of the sensors were collected by an F-7000 spectrophotometer at 25.0 ± 0.2°C. The slit widths of the fluorescence spectrophotometer for excitation and emission were both 5 nm. The maximum excitation and emission wavelength of the sensor were at 340 nm and 460 nm, respectively (see Supplementary Materials Figure S1). A fixed excitation wavelength at 340 nm was used for all of the experiments. The K_2_Cr_2_O_7_ was used as the Cr(VI) source during sensitivity studies. The concentrations of standard Cr(VI) were standardized by the AAS method.

### 2.5. Analysis of Real Water Samples

In order to assess the proposed method, lake and river water samples were obtained from Mengxi Lake and the Funan River, respectively. Mengxi Lake is in the campus of Southwest Petroleum University (Sichuan, China), and the Funan River is located in the city of Chengdu (Sichuan, China). These samples were firstly filtered through a 0.22 *μ*m cellulose membrane. To precipitate Cr(III), the pH of real water samples was adjusted to 9.0 with a certain amount of NaOH solution and then adjusted to 2.0 with HCl according to reported methods [13]. The real samples were determined by ICP-MS. CRM was also spiked in water samples, which were measured by AAS for comparisons.

## 3. Results and Discussion

### 3.1. Optimization of pH Value

**Figure 1:**
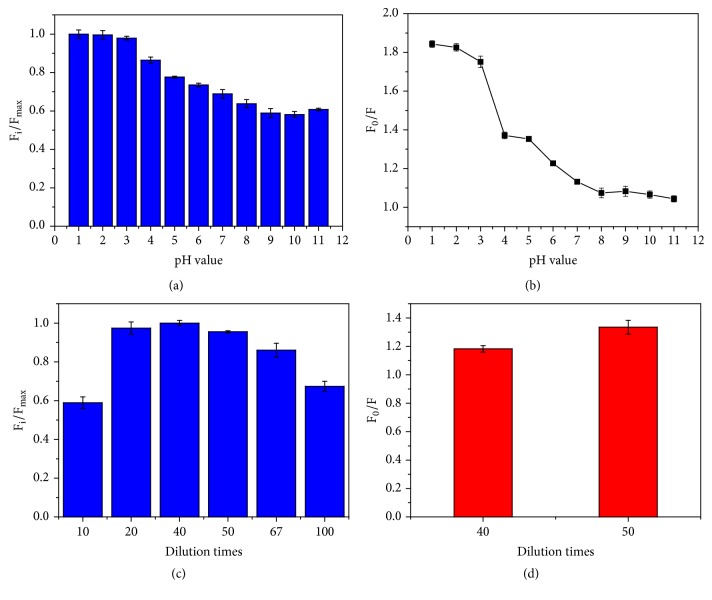
(a) The fluorescence intensity ratio (F_i_/F_max_) and (b) fluorescence responses in the presence of 20 *μ*M Cr (VI) at different pH values of F-hPEI-capped Au NPs; (c) the fluorescence intensity ratio (F_i_/F_max_) at different dilution times of F-hPEI-capped Au NPs, and (d) fluorescence responses in the presence of 4.0 *μ*M Cr (VI) for 40 and 50 dilution times of F-hPEI-capped Au NPs. The error bars represent the standard deviation of three measurements.

The pH of the solution is a very important factor for the detection of the Cr(VI) based on this sensor. In order to investigate the effect of pH, the fluorescence intensity ratio (F_i_*/*F_max_) and the sensitivity toward 20 *μ*M Cr(VI) (F_0_/F) were studied at the range of pH 1.0–11.0, as is shown in Figures 1(a) and 1(b). F_i_ and F_max_ represent the fluorescence intensity at various pH values and the maximum intensity among those pH values, respectively, while F_0_ and F are the fluorescence intensities in the absence and in the presence of Cr(VI), respectively. It is clearly observed that when the pH increased from 1 to 3, the fluorescence intensity remained stable with a slight decrease. As the pH increased further, there appeared an obvious decrease of fluorescence intensity, which reached another stable status at a pH above 8. In our previous work, it was demonstrated that the origin of the fluorescence emission for the F-hPEI-capped Au NPs was F-hPEI polymer [24]. As a result, it is suggested that the pH effect on organic polymer material actually plays a role in the change of fluorescence intensity. The branched PEI bears three protonation steps: fully deprotonated (uncharged) at pH > 10; all primary amines protonated at about pH = 7; most of amines protonated at pH < 4. Therefore, this phenomenon is probably ascribed to different protonation ratios of hPEI at various pH values. Specifically, in acidic environment, the polymer structure becomes more rigid because of the strong charge-charge repulsion and the strengthening of hydrogen bonds in acidic solution, which result in the stronger fluorescence intensity [26, 27].  Figure 1(b) exhibits the fluorescence response behaviors toward 20 *μ*M Cr(VI) showing that the sensitivity of the sensor reached its highest at pH 1.0 and 2.0. It is well-known that chromium exhibits different types of pH-dependent equilibria in aqueous solution. In pH between 1 and 6, HCrO_4_^−^ is the predominant species coexisting with Cr_2_O_7_^2−^, H_2_CrO_4_, and CrO_4_^2−^. When the pH value increases from 7 to 11, the CrO_4_^2−^ gradually becomes predominate with lower concentrations of HCrO_4_^−^. Above pH 8.0, CrO_4_^2−^ is the only species that can exist in solution [2, 28]. This response behaviors of the sensor toward Cr(VI) in different pH values were probably because, under strong acidic conditions, the positively charged sites from amino groups (for example, –NH_3_^+^ groups) interact more readily with HCrO_4_^−^ through electrostatic attraction between these two species. The decrease of the sensitivity with the increase of the pH is due to the increasing concentration of OH^−^ to compete with the HCrO_4_^−^ anion to interact with the sensor [29, 30]. It was also found that, under pH values ≤ 5, the maximum emission wavelength gradually shifted from 460 nm to 420 nm with the continuous addition of Cr(VI), while under pH values > 5, even if fluorescence had been completely quenched by Cr(VI), the maximum emission wavelength of the sensor is maintained at 460 nm. The reason for these phenomena is probably attributed to mechanism similar to the internal charge transfer (ICT) [31, 32]. In detail, a number of unreacted amine groups of hPEI on the surface of the polymer have different protonation steps under different pH values [33–35]. Meanwhile, the amine groups are responsible for the electron-donating groups in the fluorescent F-hPEI polymer [36, 37]. And the Schiff bonds may play the role of the electron acceptor. Due to the highly electron deficient of Cr(VI) in HCrO_4_^−^ anion, the electrostatic attraction between Cr(VI) and the positively charged amine groups reduces the electron-donating ability of the amine groups under acidic conditions; owing to the resulting reduction of conjugation, a blue shift of the emission spectrum is expected [32]. With the pH value increasing, the number of amines protonated gradually decreased resulting in the interaction between species containing Cr(VI) and the amine groups weakening. Thus the blue shift of the maximum emission wavelength would not occur. Considering the strong acid can inhibit the hydrolysis of metal ions, it may bring obvious interferences in assays. The pH 2.0 was chosen as the working condition. The quantum efficiency of fluorescence in pH 2.0 buffer solution was calculated to be 3.7% by using quinine sulfate as the reference.

### 3.2. Optimization of the Dilution Times

In order to improve the performance of the sensor, the dilution times for the F-hPEI-capped Au NPs in buffer solution during the measurement procedure have also been studied. As shown in Figure 1(c), the fluorescence intensity first increased with increasing dilution times, reaching a maximum at 40-fold dilution. This is probably because the diluted solution can avoid any self-absorption effects. However, increasing the dilution times further, the fluorescence intensity decreased, attributed to the lower concentration of probes. The fluorescence intensities of the 20-fold and 50-fold dilution amounts were nearly the same, which were slightly lower than the maximum. As gold is a precious metal, the dilution amounts of 40-fold and 50-fold were chosen for the sensitivity study for the economic reasons. As Figure 1(d) shows, the 50-fold dilution showed higher sensitivity than that of 40-fold. Therefore, the sensor of a 50-fold dilution was chosen for analysis.

### 3.3. Performance of Cr(VI) Sensor

**Figure 2:**
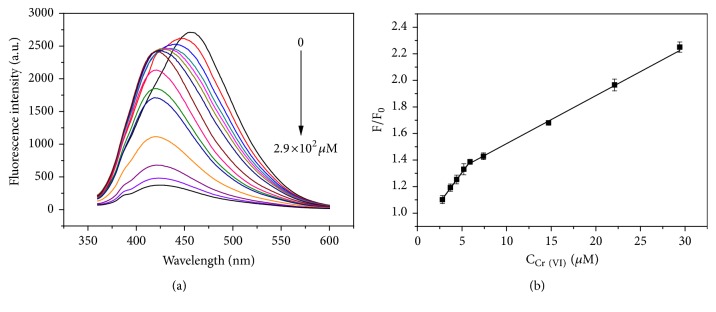
(a) Fluorescence spectra of F-hPEI-capped Au NPs with the addition of different concentrations of Cr (VI) ranging from 0 *μ*M to 2.9×10^2^ *μ*M. (b) The corresponding calibration curve for this sensor over the range from 2.8 *μ*M to 29 *μ*M. The error bars represent the standard deviation of three measurements.

To evaluate the sensitivity of the sensor for Cr(VI) determination, the emission spectra of the F-hPEI-capped Au NPs upon different concentrations of Cr(VI) were measured by a fluorometer. As Figure 2(a) shows, the fluorescence emission of the sensor was first quenched and was accompanied by a blue shift of the emission peak from 460 nm to 420 nm. Then, the blue shift of the emission peak stopped, and the intensity continued to decrease upon increasing the concentration of Cr(VI). The values of F_0_/F at 460 nm of each spectrum were plotted with the Cr(VI) concentrations in the range of 0–29 *μ*M. The calibration curve (Figure 2(b)) displayed two linear ranges, with the lower Cr(VI) concentrations ranging from 2.8 *μ*M to 5.9 *μ*M; the equation was* F*_*0*_*/F *= 9.1×10^−2^*C* + 0.8524 (R^2^ = 0.9988), where* C* was the concentration of Cr(VI) (*μ*M). The LOD can be calculated following the IUPAC criterion, LOD = 3 S_0_/*m*, where “*m*” is the slope of the calibration equation, and “S_0_” is the RSD of the 11 blank signals. Similarly, the limit of quantification (LOQ) was calculated by LOQ = 10 S_0_/*m*. The LOD and LOQ obtained by above equations were 0.63 *μ*M and 2.0 *μ*M, respectively. The LOD value of 0.63 *μ*M was lower than the provisional guideline value of total chromium in groundwater by WHO [25]. Furthermore, this concentration range contained not only the changes of the fluorescence intensity but also a blue shift of the emission peak, which could provide a more precise visual method, while at higher concentration range from 5.9 *μ*M to 29 *μ*M, a relatively low sensitivity was obtained by the calibration curve* F*_*0*_*/F* = 3.6×10^−2^*C* + 1.158 (R^2^ = 0.9914). This concentration range only showed a decrease of the intensity, without the changes in the emission wavelength. The two linear trends toward Cr(VI) detection were corresponding to two phenomena: at lower concentration range from 2.8 *μ*M to 5.9 *μ*M, the fluorescence intensity decreased with the emission wavelength shifting from 460 nm to 420 nm; at concentration range higher than 5.9 *μ*M, the fluorescence intensity decreased with a fixed blue shift emission wavelength at 420 nm. In section of* Optimization of pH value*, we have ascribed the blue shift of the emission wavelength to the reduction of conjugation caused by electrostatic attraction between Cr(VI) and the positively charged amine groups. Therefore, the two linear trends were probably because the conjugation of the fluorophore gradually decreased with the addition of low concentration of Cr(VI), while when concentration of Cr(VI) reached 5.9 *μ*M, the conjugation decreased to a minimum which would not change even if the concentration exceeded 5.9 *μ*M. The precision for five replicate detections of 3.7 *μ*M and 15 *μ*M was 3.6% and 1.3% (RSD), respectively. The results indicate the sensor has a good linear relationship, sensitivity, and precision for Cr(VI).

**Figure 3:**
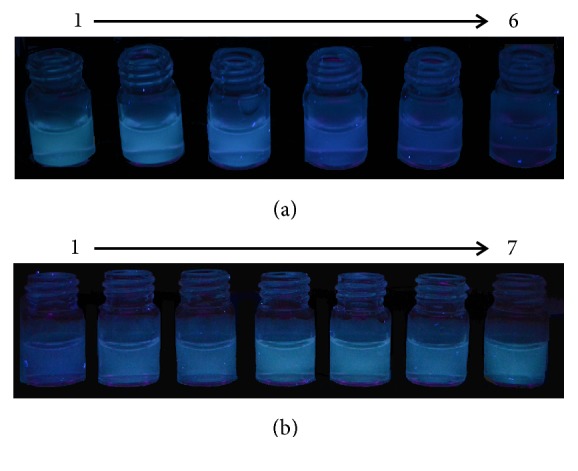
(a) Fluorescence photograph of F-hPEI-capped Au NPs upon different concentrations of Cr(VI): (1) 0, (2) 4.0 (3) 8.0, (4) 32, (5) 40, and (6) 80 *μ*M. (b) Fluorescence photograph of F-hPEI-capped Au NPs upon different concentrations of Fe(II): (1) 0, (2) 2.9×10^2^, (3) 5.8×10^2^, (4) 8.6×10^2^, (5) 1.2×10^3^, (6) 1.4×10^3^ *μ*M, in the presence of 80 *μ*M Cr(VI), and (7) the blank solution without Cr (VI) and Fe (II).

Viewing the oxidization of the potassium dichromate under acidic conditions, the fluorescence intensity of the quenched sensor may be recovered by some reducing agents. Herein, we selected the Fe(II) ion to study the recovery of the sensor. The reaction time for the Fe(II) was set as 10 min. After being quenched by 160 *μ*M Cr(VI) and the addition of increasing concentrations of Fe(II), the partly quenched fluorescence intensity of the sensor increased gradually to 61% of the fluorescence intensity, with a red shift of the emission peak from 420 nm to 440 nm (Figure S2(a)). However, when the concentration of Fe(II) was increased from 1.2×10^3^ *μ*M to 1.4×10^3^ *μ*M, the fluorescence intensity did not increase further and was accompanied by a blue shift of the emission peak from 440 nm to 420 nm (Figure S2(b)). Furthermore, it was found that when reducing the concentration of the Cr(VI) to 80 *μ*M and 40 *μ*M, the sensors' fluorescence intensities could be recovered by Fe(II), at 86% and 97%, respectively, with the fluorescence emission shifting to about 460 nm (Figures S2(c)-S2(d)). The detection of Fe(II) was investigated based on a solution that had been quenched by 160 *μ*M Cr(VI). In Figure S3(a), the spectra demonstrated the changes when addition of increasing concentrations of Fe(II). In Figure S3(a), the spectra demonstrated the changes with the addition of increasing concentrations of Fe(II). The fluorescence intensity of the sensor at 440 nm and the concentration of the Fe(II) accorded with the following equation (Figure S3(b)), -*F/F*_*0*_ = *a*lg⁡*C* + *b*, where* F* and* F*_*0*_ were the fluorescence intensity in the presence and absence of Fe(II), respectively.* C* was the concentration of the Fe(II) ion (*μ*M). The response curve for the Fe(II) determination exhibited a linear relationship at concentrations from 1.4×10^2^ *μ*M to 1.2×10^3^ *μ*M; the linear equation was* -F/F*_*0*_ = 1.0 lg* C* + 0.9654 (R^2^ = 0.9998). The results demonstrated that the interaction between Fe(II) ions and the quenched sensors was quantitative, and the quenched sensor could be used as a “turn-on” Fe(II) sensor. The fluorescence intensity quenched (Figure 3(a)) and recovered (Figure 3(b)) by the addition of Cr(VI) and Fe(II) could also be observed by the naked eye with the aid of a UV lamp. The obvious fluorescence intensity changes under a UV lamp exhibit a facile method to determine these two metal ions.

### 3.4. Exploration of the “Turn-Off” and “Turn-On” Mechanism

**Figure 4:**
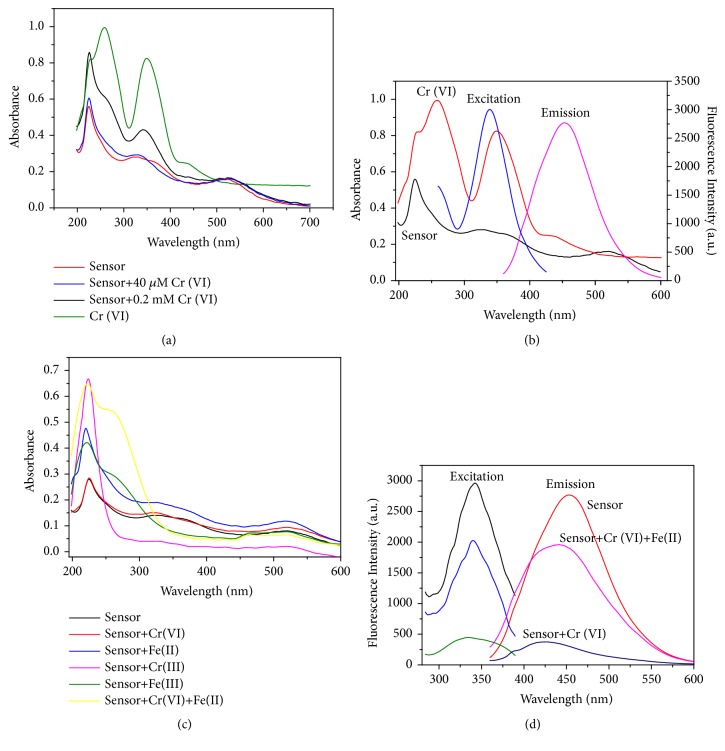
(a) UV-Vis absorption spectra of Cr(VI) and the sensor before and after the addition of Cr(VI). (b) Fluorescence spectra of the sensor and UV-Vis absorption spectra of the Cr(VI) and the sensor. (c) UV-Vis absorption spectra of the sensor before and after the addition of different kinds of species. (d) The excitation and emission spectra of the sensor before and after the addition of different kinds of species.

In order to investigate the “turn-off” mechanism, the UV-Vis absorption spectra and fluorescence excitation and emission spectra of the sensor with different concentrations of Cr(VI) were collected. In Figure 4(a), the F-hPEI-capped Au NPs exhibited three absorption peaks at 224 nm, 330 nm, and 520 nm. The first two absorption peaks are ascribed to the F-hPEI [20], while the peak at 520 nm originates from the surface plasmon resonance of the Au NPs. Upon continuous addition of Cr(VI), the absorption spectra of the F-hPEI-capped Au NPs were almost unchanged until about 74 *μ*M, indicating there was nonfluorescent complex formation between Cr(VI) and the sensor. Because the fluorescence emission can be ascribed to n*←π∗* transitions of C=N bonds, the changeless absorption spectra indicated that the interaction between Cr(VI) and the sensor only affected the excited state *π∗* and hence no variation in the absorbance spectra is expected [38]. Furthermore, the average fluorescence lifetime before and after addition of Cr(VI) was 2.2 ns and 1.7 ns, respectively, which illustrates the sensor for the Cr(VI) determination was also based on the principle of dynamic quenching [38]. TEM image illustrated the addition of Cr(VI) did not cause the obvious change or aggregation of the F-hPEI-capped Au NPs (Figure S4), which further eliminates Au NPs aggregation-induced self-quenching and the possibility of direct bonding between Cr(VI) and Au NPs. Thus, the absorption band of surface plasmon resonance of Au NPs at 520 nm was not shifted or changed.

As shown in Figure 4(b), the K_2_Cr_2_O_7_ exhibited broad absorption at 260, 360, and 440 nm. The broad absorption spectrum of K_2_Cr_2_O_7_ exhibited wide and precise overlapping with the excitation and emission bands of the sensor, indicating both the excitation and emission lights are filtered by Cr(VI). When the excitation light and emission light reached the solution containing F-hPEI-capped Au NPs and Cr(VI), Cr(VI) could absorb most of the light, and the fluorescence intensity of sensor decreased. As a result, the quenching process also exhibited the inner filter effect. In summary, the quenching mechanism is mainly attributed to the following two reasons: Cr(VI) which is bound to the free amine groups of polyethyleneimine that surround Au NPs by electrostatic attraction and the charge transfer from amine groups to Cr(VI) reducing the electron-donating ability of the amine groups; the absorption of excitation and emission fluorescence of sensor by Cr(VI).

**Scheme 1:**
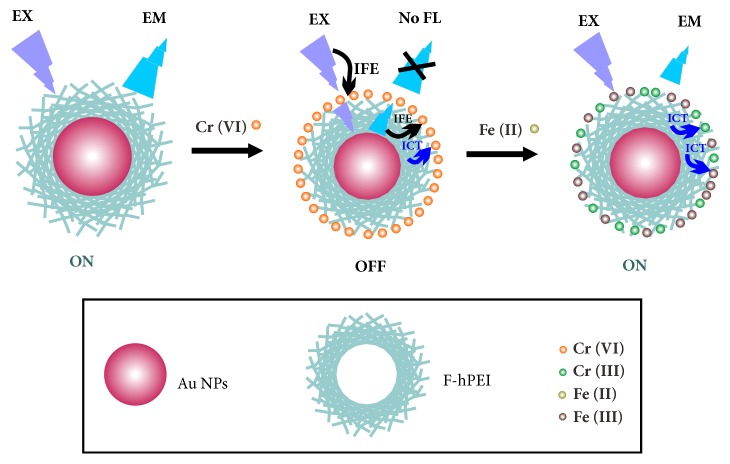
Proposed “turn-off” and “turn-on” mechanism based on the fluorescent sensor.

To confirm the redox reaction during the “turn-on” process, UV-Vis absorption spectra of the sensor with different species were measured. The UV-Vis absorption spectrum after the addition of Fe(II) is shown in Figure 4(c). It was demonstrated that, after the “turn-on” reaction, a broad and strong absorption band around 220 nm with a shoulder peak at 260 nm appeared. In order to investigate the spectrum, the UV-Vis spectra of the F-hPEI-capped Au NPs containing only Cr(III), Fe(III), and Fe(II), separately, were collected. The UV-Vis spectrum of the sensor containing Fe(III) was similar to that of the sensor containing Cr(VI) and Fe(II), which illustrated that Fe(III) is present in the “turn-on” solution. Thus, it has been proven that redox reactions have occurred between Cr(VI) and Fe(II). Once the Fe^2+^ was added to the Cr(VI), the HCrO_4_^−^ anion and Fe^2+^ were changed into Cr^3+^ and Fe^3+^, respectively. Therefore, the electrostatic interaction had been weakened due to the presence of similar charge, which could not reduce the electron-donating ability of the amine groups under acid environment, owing to the resulting raise of conjugation. Therefore, the red shift occurred. Also, the inner filter effect disappeared due to the absence of overlapping of the excitation and emission of the fluorescence. As a result, the quenched fluorescence could be recovered. It is well-known that the amine groups of PEI can adsorb some metal ions via coordination [39–41]. The strong absorption bands at 230 nm and 260 nm appeared in the spectra indicating the sensor could form coordination complexes with Cr(III) and Fe(III). According to the results of the interference study (see* Study of Interference*), the high concentration of Fe(III) ions and Cr(III) ions can interfere with the detection of Cr(VI) probably due to ICT mechanisms. Therefore, when the concentration of Cr(VI) is high, the fluorescence emission intensity can only be partly recovered because of the interference from the Fe(III) and Cr(III). Furthermore, the excitation and emission spectra of the “turn-off” and “turn-on” systems were also collected (Figure 4(d)). The results exhibited that the maximum excitation wavelength did not shift or change, which indicated that no ground-state complex had been formed in the quenching and recovering processes. Therefore, the static quenching mechanism could be ruled out. The proposed “turn-off” mechanism for Cr(VI) and “turn-on” mechanism for Fe(II) are depicted in Scheme 1.

### 3.5. Study of Interference

In the present study, interferences of foreign species on the determination of 7.4 *μ*M Cr(VI) were investigated. The tolerance ratios (C_chemical  species_/C_Cr  (VI)_) when interference concentration was varying the analytical signal by 5% are presented in Table S2 (see Supplementary Materials). This table demonstrated most species almost did not affect the determination of Cr(VI), with the exceptions of the Fe(II) and I^−^ ions, which exhibited a few interferences with the determination of Cr(VI). The interferences from the Fe(II) and I^−^ ions were due to their reducing abilities, which could react with Cr(VI) under acidic condition. Therefore, Fe(II) and I^−^ ions caused interference by reducing the Cr(VI) to Cr(III). The tolerance ratio for Fe(III) was 2.5, which illustrated that unusually excessive Fe(III) ions in the solution would have an obvious impact on the sensors. The interference mainly originates from the strong binding strength between Fe(III) ions and amine groups on the sensors. Although the tolerance ratio for Cr(III) was 50, the strong absorption bands at 230 nm (Figure 4(c) pink line) also indicated the existence of an interaction between Cr(III) and the sensors; these results illustrated the threshold of detecting Fe(II) based on the sensor, as the excessive reactants Fe(III) and Cr(III) interfere with the determination. Considering the tolerance ratio for Fe(III) is much lower than that of Cr(III), the Fe(III) ions are the main source of interference. Also, because the concentration of Fe(II) is much higher than that of Cr(VI) in the recovery procedure, the intensity of fluorescence that could be recovered depends on the concentration of Cr(VI).

### 3.6. Detection of Cr (VI) in Real Water Samples

For testing the reliability and accuracy of the proposed method, the sensor was applied for real analysis of Cr(VI) in lake water and river water. The concentrations of Cr(VI) determined by ICP-MS in these two samples were less than 10 ppb, which was far below the detection limit of the proposed method. Therefore, the concentrations of the water samples could be ignored. These water samples, spiked with different standard Cr(VI) solutions (3.0 *μ*M, 11.0 *μ*M, and 22.0 *μ*M) were analyzed.  Table 1 displays that the proposed method had the satisfactory agreements with the AAS. The quantitative recovery range was from 96.7% to 105%, which illustrated the proposed method could give reliable analysis results for Cr(VI) in real applications.

## 4. Conclusions

In conclusion, a new method based on F-hPEI-capped Au NPs for fluorescent detection and speciation of Cr(VI) has been demonstrated. The sensors demonstrate high sensitivity and satisfying selectivity for determination of Cr(VI). The result shows the sensor has been successfully applied to detect Cr(VI) in real samples. Furthermore, Fe(II) ions can recover the quenched fluorescence intensity. However, according to the interference study, Fe(III) and Cr(III) ions can act as reactants and bring a significant influence to the recovery process. As a result, the recovered intensity is dependent on the concentration of Cr(VI). The quenching mechanism is based on the combined effects of the IFE and ICT, while the recovering mechanism is based on the redox reaction between Cr(VI) and Fe(II) ion under acid environments. Given that this sensor is synthesized facilely and the method is accurate and reliable, we envision that it is very promising for application in practice.

## Figures and Tables

**Table 1:** Determination of Cr (VI) in real water samples.

Sample	Added	Proposed method found	Recovery	RSD	AAS found
	(*μ*M)	(*μ*M)^a^	(%)	(%)	(*μ*M)
Lake water	0	Not detected	-	-	0.033^b^
	3.00	3.12	104	3.56	3.00
	12.0	11.7	97.6	2.22	11.9
	22.0	22.2	101	1.03	22.8
River water	0	Not detected	-	-	0.073^b^
	3.00	2.90	96.7	2.32	3.00
	12.0	12.6	105	2.26	12.9
	22.0	23.2	105	2.65	21.7

a: average value of four measurements. b: the samples are determined by ICP-MS.

## Data Availability

Data are available upon request from Na Wang, wangna@swpu.edu.cn.

## Supplementary Materials

Supplementary Materials Table S1: Comparison of the present approach with other reported methods for the detection of Cr (VI) Table S2: Investigation of potential interferents in determination of Cr(VI) at 7.4 *μ*M concentration. Figure S1: The excitation and emission fluorescence spectra of the F-hPEI capped Au NPs. Figure S2: (a) (A) (B) represent the fluorescence emission spectra of the F-hPEI-capped Au NPs in the absence and presence of 160 *μ*M Cr(VI), line C is the sensor containing 160 *μ*M Cr(VI) and 1.2×10^3^*μ*M Fe(II) ion. (b) The fluorescence emission spectra of the F-hPEI-capped Au NPs containing 160 *μ*M Cr(VI) in the presence of 1.2×10^3^*μ*M and 1.4×10^3^*μ*M Fe(II) ion. (c) (A) (B) represent the fluorescence emission spectra of the F-hPEI-capped Au NPs in the absence and presence of 80 *μ*M Cr(VI), line C is the sensor containing 80 *μ*M Cr(VI) and 1.4×10^3^*μ*M Fe(II) ion. (d) (A) (B) represent the fluorescence emission spectra of the F-hPEI-capped Au NPs in the absence and presence of 40 *μ*M Cr(VI), line C is the sensor containing 40 *μ*M Cr(VI) and 1.4×10^3^*μ*M Fe(II) ion. Figure S3: (a) Fluorescence spectra of F-hPEI capped Au NPs containing 160 *μ*M Cr(VI) with the addition of different concentrations of Fe(II) ranging from 0 *μ*M to 1.2×10^3^*μ*M. The concentrations corresponding to the spectra from bottom to top are 1.4×10^2^*μ*M, 2.9×10^2^*μ*M, 5.8×10^2^*μ*M, 8.6×10^2^*μ*M, 1.2×10^3^*μ*M. (b) The corresponding calibration curve for this sensor over the range from 1.4×10^2^*μ*M to 1.2×10^3^*μ*M. The error bars represent the standard deviation of three measurements. Figure S4: The TEM images of F-hPEI capped Au NPs (a) before and (b) after addition of Cr(VI). The insets are the TEM images with high magnification.
