# Supplementary material for: Fluorescent Sensors Based on Organic Polymer-Capped Gold Nanoparticles for the Detection of Cr(VI) in Water
Source: Int J Anal Chem. 2019 Feb 3;2019:1756014. doi: 10.1155/2019/1756014 (PMC6378049; doi:10.1155/2019/1756014)
Supplement: Supplementary Materials — Table S1: Comparison of the present approach with other reported methods for the detection of Cr (VI) Table S2: Investigation of potential interferents in determination of Cr(VI) at 7.4 μM concentration. Figure S1: The excitation and emission fluorescence spectra of the F-hPEI capped Au NPs. Figure S2: (a) (A) (B) represent the fluorescence emission spectra of the F-hPEI-capped Au NPs in the absence and presence of 160 μM Cr(VI), line C is the sensor containing 160 μM Cr(VI) and 1.2×10^3^μM Fe(II) ion. (b) The fluorescence emission spectra of the F-hPEI-capped Au NPs containing 160 μM Cr(VI) in the presence of 1.2×10^3^μM and 1.4×10^3^μM Fe(II) ion. (c) (A) (B) represent the fluorescence emission spectra of the F-hPEI-capped Au NPs in the absence and presence of 80 μM Cr(VI), line C is the sensor containing 80 μM Cr(VI) and 1.4×10^3^μM Fe(II) ion. (d) (A) (B) represent the fluorescence emission spectra of the F-hPEI-capped Au NPs in the absence and presence of 40 μM Cr(VI), line C is the sensor containing 40 μM Cr(VI) and 1.4×10^3^μM Fe(II) ion. Figure S3: (a) Fluorescence spectra of F-hPEI capped Au NPs containing 160 μM Cr(VI) with the addition of different concentrations of Fe(II) ranging from 0 μM to 1.2×10^3^μM. The concentrations corresponding to the spectra from bottom to top are 1.4×10^2^μM, 2.9×10^2^μM, 5.8×10^2^μM, 8.6×10^2^μM, 1.2×10^3^μM. (b) The corresponding calibration curve for this sensor over the range from 1.4×10^2^μM to 1.2×10^3^μM. The error bars represent the standard deviation of three measurements. Figure S4: The TEM images of F-hPEI capped Au NPs (a) before and (b) after addition of Cr(VI). The insets are the TEM images with high magnification. [file 1756014.f1.pdf]

Supplementary Materials

Fluorescent sensors based on organic polymer capped gold nanoparticles for the detection of Cr(VI) in water

Na Wang*, Liangchen Wang, Hong Yang, Tingting Xiong, Shangping Xiao, Jiawen Zhao and Weiping Du

School of Chemistry & Chemical Engineering, Southwest Petroleum University, Chengdu 610500, Sichuan, PR China

*Corresponding author. Tel: +86 028 8303 7341

Fax: +86 028 8303 7305

E-mail address: wangna@swpu.edu.cn

Table S1. Comparison of the present approach with other reported methods for the detection of Cr (VI) in aqueous solution.

Method	Probe	LOD	Linearity	Ref
AAS	-	Cr(III): 1.9 μg/L;<br>Cr(VI): 2.3 μg/L;	-	1
AAS	-	Both: 0.01: μg/L		2
ICP-MS	-	Both: 0.03: μg/L	-	3
ICP-MS	-	Both: 0.2: μg/L	-	4
Electrochemical	Au-NP-modified SPE electrode	Cr (VI): 0.5 μg/L	10 μg/L -5 mL/L	5
Electrochemical	Ti/TiO_2NT/Au electrode	Cr(VI): 0.03 μM	0.1-105 μM	6
Colorimetry	Ag_{core} - Au_{shell}	Cr(VI): 1×10^{-8} M	1×10^{-8} - 8×10^{-6} M	7
Colorimetry	Au nanorods	Cr(VI): 8.8×10^{-8} M	0.1-20 μM	8
Fluorescence	PEI-Ag NPs	Cr(VI): 0.04 nM	0.1 nM - 3.0 μM	9
Fluorescence	11- MUA-AuNCs	Cr(III): 26 nM;	25 nM - 10 μM	10
Fluorescence	Carbon dots	Cr(VI): 0.30 μM	0.01-50 μM	11
This method	F-PEI-capped Au NPs	Cr(VI): 0.6 μM	2.8 μM -5.9 μM<br>5.9 μM -29 μM	This work

Table S2. Investigation of potential interferents in determination of Cr(VI) at 7.4 μM concentration.

Tolerance (Interferent/ Cr (VI) molar ratio	Foreign species
100	Na^+, K^+, Cl^-, ClO_3^-, Ca^{2+}, PO_4^{3-}, HPO_4^{2-}, H_2PO^{4-}, HCO_3^-, SO_4^{2-}, Mg^{2+}, Mn^{2+}, NO_3^-, Ni^{2+}
50	Cr^{3+}, Cu^{2+}, Br^-,
25	Pb^{2+}, Zn^{2+},
12.5	Ag^+
5	Al^{3+}
2.5	Cd^{2+}, Fe^{3+}
1	Fe^{2+}, I^-

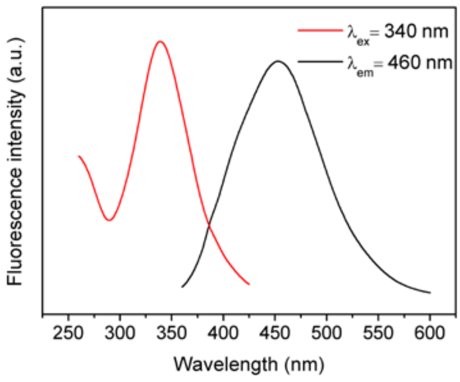

Figure S1. The excitation and emission fluorescence spectra of the F-hPEI capped Au NPs.

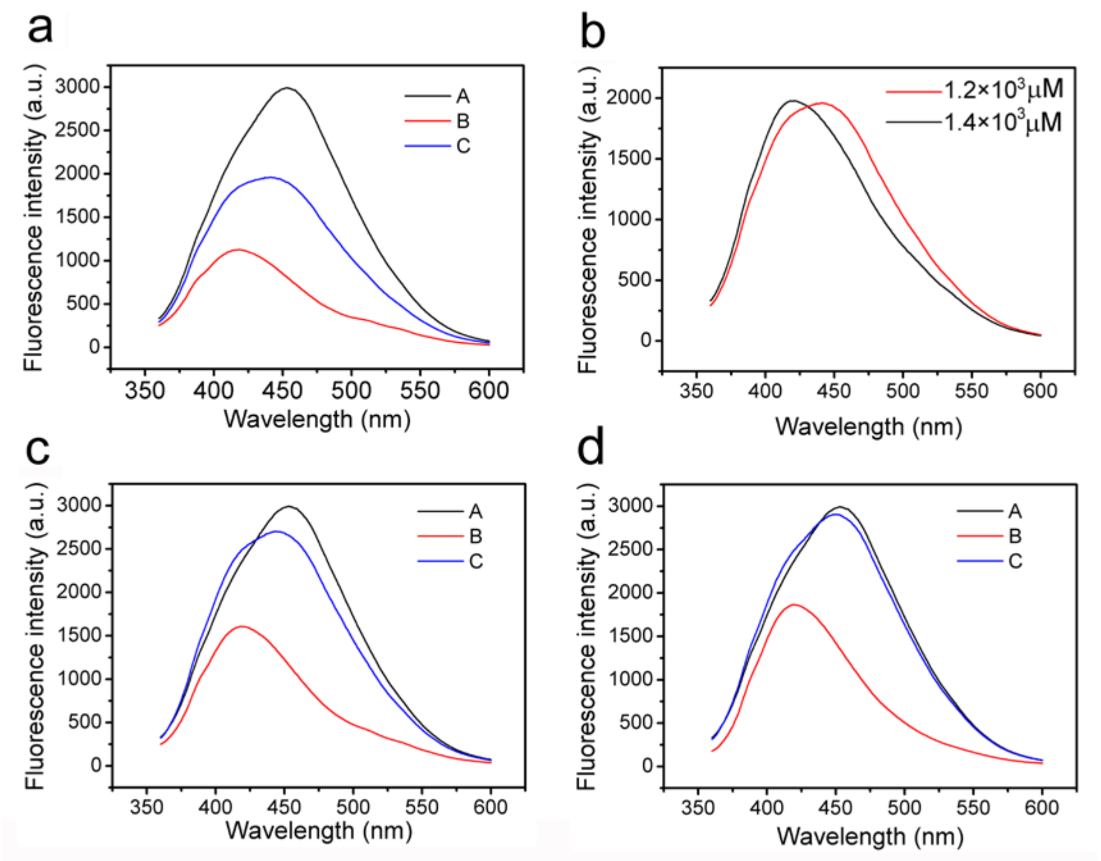

Figure S2. (a) (A) (B) represent the fluorescence emission spectra of the F-hPEI-capped Au NPs in the absence and presence of 160 μM Cr(VI), line C is the sensor containing 160 μM Cr(VI) and 1.2×10^3 μM Fe(II) ion. (b) The fluorescence emission spectra of the F-hPEI-capped Au NPs containing 160 μM Cr(VI) in the presence of 1.2×10^3 μM and 1.4×10^3 μM Fe(II) ion. (c) (A) (B) represent the fluorescence emission spectra of the F-hPEI-capped Au NPs in the absence and presence of 80 μM

Cr(VI), line C is the sensor containing 80 μM Cr(VI) and 1.4×10^3 μM Fe(II) ion. (d) (A) (B) represent the fluorescence emission spectra of the F-hPEI-capped Au NPs in the absence and presence of 40 μM Cr(VI), line C is the sensor containing 40 μM Cr(VI) and 1.4×10^3 μM Fe(II) ion.

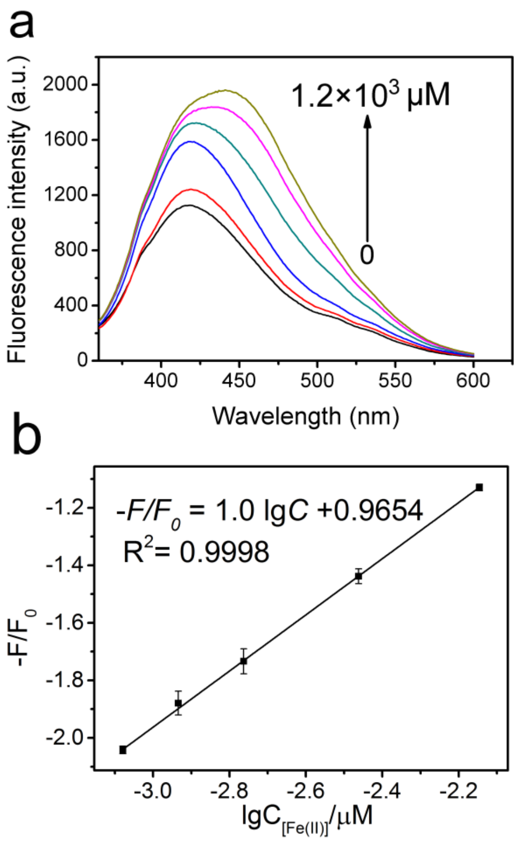

Figure S3. (a) Fluorescence spectra of F-hPEI capped Au NPs containing 160 μM Cr (VI) with the addition of different concentrations of Fe(II) ranging from 0 μM to 1.2×10^3 μM. The concentrations corresponding to the spectra from bottom to top are 1.4×10^2 μM, 2.9×10^2 μM, 5.8×10^2 μM, 8.6×10^2 μM, 1.2×10^3 μM. (b) The corresponding calibration curve for this sensor over the range from 1.4×10^2 μM to 1.2×10^3 μM. The error bars represent the standard deviation of three measurements.

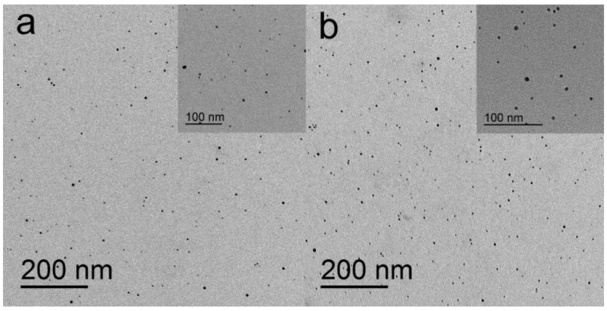

Figure S4. The TEM images of F-hPEI capped Au NPs (a) before and (b) after addition of Cr(VI). The insets are the TEM images with high magnification.

References

1 H.F. Maltez, E. Carasek, Chromium speciation and preconcentration using zirconium(IV) and zirconium(IV) phosphate chemically immobilized onto silica gel surface using a flow system and F AAS, *Talanta*. 65 (2005) 537-542.

2 A. Sahayam, Speciation of Cr(III) and Cr(VI) in potable waters by using activated neutral alumina as collector and ET–AAS for determination, *Analytical and Bioanalytical Chemistry*. 372 (2002) 840-842.

3 H.-J. Wang, X.-M. Du, M. Wang, T.-C. Wang, H. Ou-Yang, B. Wang, M.-T. Zhu, Y. Wang, G. Jia, W.-Y. Feng, Using ion-pair reversed-phase HPLC ICP-MS to simultaneously determine Cr(III) and Cr(VI) in urine of chromate workers, *Talanta*. 81 (2010) 1856-1860.

4 Z. Chen, M. Megharaj, R. Naidu, Speciation of chromium in waste water using ion chromatography inductively coupled plasma mass spectrometry, *Talanta*. 72 (2007) 394-400.

5 G. Liu, Y.-Y. Lin, H. Wu, Y. Lin, Voltammetric Detection of Cr(VI) with Disposable Screen-Printed Electrode Modified with Gold Nanoparticles, *Environmental Science & Technology*. 41 (2007) 8129-8134.

6 W. Jin, G. Wu, A. Chen, Sensitive and selective electrochemical detection of chromium(vi) based

on gold nanoparticle-decorated titania nanotube arrays, *Analyst*. 139 (2014) 235-241.

7 J. Xin, F. Zhang, Y. Gao, Y. Feng, S. Chen, A. Wu, A rapid colorimetric detection method of trace Cr(VI) based on the redox etching of Agcore–Aushell nanoparticles at room temperature, *Talanta*. 101 (2012) 122-127.

8 F.-M. Li, J.-M. Liu, X.-X. Wang, L.-P. Lin, W.-L. Cai, X. Lin, Y.-N. Zeng, Z.-M. Li, S.-Q. Lin, Non-aggregation based label free colorimetric sensor for the detection of Cr(VI) based on selective etching of gold nanorods, *Sensors and Actuators B: Chemical*. 155 (2011) 817-822.

9 J.R. Zhang, A.L. Zeng, H.Q. Luo, N.B. Li, Fluorescent silver nanoclusters for ultrasensitive determination of chromium(VI) in aqueous solution, *Journal of Hazardous Material*. 304 (2016) 66-72.

10 J. Sun, J. Zhang, Y. Jin, 11-Mercaptoundecanoic acid directed one-pot synthesis of water-soluble fluorescent gold nanoclusters and their use as probes for sensitive and selective detection of Cr^{3+} and Cr^{6+}, *Journal of Materials Chemistry C*. 1 (2013) 138-143.

11 M. Zheng, Z. Xie, D. Qu, D. Li, P. Du, X. Jing, Z. Sun, On–Off–On Fluorescent Carbon Dot Nanosensor for Recognition of Chromium(VI) and Ascorbic Acid Based on the Inner Filter Effect, *ACS Applied Materials & Interfaces*. 5 (2013) 13242-13247.
